# Exploring the causal relationship between sedentary behavior and cardiovascular disease: A two-sample Mendelian randomization analysis

**DOI:** 10.1097/MD.0000000000044543

**Published:** 2025-09-12

**Authors:** Ye Cao, Qing Hao, Nan Xia, Yi Feng

**Affiliations:** a Department of Cardiology, Renmin Hospital, Hubei University of Medicine, Shiyan, Hubei, PR China; b Department of Pulmonary and Critical Care Medicine, Renmin Hospital, Hubei University of Medicine, Shiyan, Hubei, PR China.

**Keywords:** causal effect, genome-wide association study, Mendelian randomization, sedentary behavior, cardiovascular disease

## Abstract

Cardiovascular disease (CVD) is the primary cause of death globally. However, the causal association between sedentary behavior (SB) and CVD outcomes remains unknown. Genome-wide association study summary statistics for SB and CVD outcomes were collected from the FinnGen and UK Biobank. Mendelian randomization analyses were applied to explore the causal effects of SB on CVD outcomes by selecting single nucleotide polymorphisms as instrumental variables. Genetically predicted leisure screen time was significantly associated with increased risks of cardiovascular disease (odds ratio [OR] = 1.13, 95% confidence interval [CI]: 1.02–1.24), coronary artery disease (OR = 1.18, 95% CI: 1.08–1.29), heart failure (OR = 1.10, 95% CI: 1.02–1.19), and ischemic stroke (OR = 1.14, 95% CI: 1.04–1.25). Time spent watching TV was also positively associated with cardiovascular disease (OR = 1.32, 95% CI: 1.02–1.72). No significant associations were observed for computer use or driving. This Mendelian randomization study suggests a causal relationship between both genetically predicted leisure screen time and television viewing with increased cardiovascular disease risk, particularly coronary artery disease. No causal effects were observed for using computer or driving. These findings underscore the public health relevance of targeting specific sedentary behaviors in strategies to prevent cardiovascular diseases.

## 1. Introduction

Cardiovascular disease (CVD) is the primary cause of death globally, making CVD prevention an international public health priority.^[1,2]^ Sedentary behavior (SB) is defined as any waking behavior that involves an energy expenditure ≤ 1.5 metabolic equivalents of task when sitting, reclining, or lying.^[3–5]^ According to the 2018 Physical Activity Guidelines Advisory Committee scientific report, there is substantial evidence of a positive dose–response relationship between SB and CVD prevalence and mortality.^[6]^ Similarly, observational studies indicate that SB is associated with higher risks of cardiovascular diseases and events.^[7,8]^

However, the epidemiologic evidence is inconsistent. A 2019 meta-analysis including 36,383 participants with a median follow-up of 5.8 years found that sedentary time >9.5 h/d was associated with a significantly increased risk of all-cause mortality.^[9]^ In addition, a meta-analysis in 2018 found that the relationship between CVD risk and sedentary time is nonlinear; thresholds of >6 h/d for total sedentary time and >4 h/d for television viewing were associated with significantly higher CVD risk.^[10]^ Thresholds differed across studies. Moreover, TV-viewing time accounts for just a minor percentage of the waking day. Compared with TV viewing, a systematic review found no association between occupational sitting and adverse health outcomes.^[11]^

Although accumulating observational evidence has demonstrated associations between SB and CVD, establishing a definitive causal relationship has been methodologically challenging. Conventional observational designs are inherently limited by residual confounding factors and potential reverse causation bias. For instance, individuals with subclinical or existing cardiovascular conditions may be more likely to adopt sedentary lifestyles, while unmeasured factors such as dietary patterns, socioeconomic status, and other behavioral characteristics may confound the observed associations between SB and CVD outcomes.

While recent observational studies have employed more rigorous methodologies and larger sample sizes, these fundamental limitations persist. The causal nature of the SB–CVD relationship, therefore, remains uncertain under conventional epidemiologic approaches. Mendelian randomization (MR) offers a powerful alternative methodology for causal inference by utilizing genetic variants as instrumental variables (IVs).^[12]^ This approach is particularly valuable in CVD research for 2 key reasons. First, since genetic variants are randomly allocated during meiosis prior to disease onset, MR analyses are theoretically protected from reverse causation. Second, the fixed nature of genetic variants makes them less susceptible to confounding by lifestyle or environmental factors that typically bias observational studies.^[13]^ In this study, a 2 -sample MR research design was used to explore the potential causality between SB and CVD outcomes with the use of extensive genome-wide association study (GWAS) summary data.

## 2. Materials and methods

### 2.1. Study design

This study adhered to the STROBE-MR (strengthening the reporting of observational studies in epidemiology using Mendelian randomization) guidelines.^[14]^ Robust causal inference from MR requires three core assumptions. First, the exposure—SB—is strongly associated with the genetic IVs. Second, the possible confounders such as body mass index, lipoproteins, educational attainment, smoking status, dietary patterns, comorbidities, and others have nothing to do with the genetic IVs. Third, the genetic IVs affect the outcome—CVD outcomes—only through SB, with no alternative pathways.^[15,16]^

### 2.2. Data sources

While the ideal measure of SB would capture all waking activities performed in a sitting or reclining posture with low energy expenditure, current GWAS datasets lack comprehensive genetic instruments for total sedentary time. Therefore, this study selected 4 traits as proxies for SB: leisure screen time (LST), time spent watching television, time spent using computer, and time spent driving. These traits represent the most consistently measured and heritable components of sedentary behavior in large-scale population cohorts. LST summary statistics were obtained from a large-scale GWAS meta-analysis of 51 cohorts, where LST was defined as time spent on recreational screen-based activities—including television viewing, video gaming, and computer use—with standardized measurements across participating studies.^[17]^ GWAS summary statistics for time spent watching TV, using computer, and driving were derived from the UK Biobank. The basic information about the GWAS database of the exposure can be seen in Table 1. The outcomes include CVD, coronary artery disease (CAD), heart failure (HF), atrial fibrillation (AF), and ischemic stroke (IS).^[12]^ For the same circumstance, we acquired summary-level data for the outcome events from the GWAS database. Table 2 shows the fundamental information about the GWAS database of the outcome. Notably, we acknowledge partial sample overlap between exposure and outcome datasets, primarily due to the inclusion of UK Biobank participants in both.

**Table 1:** Basic information about the GWAS database of the exposure.

Exposure	GWAS-ID	Year	Population	Number of SNPs	Sample size
Leisure screen time	ebi-GCST90104339	2022	European	15,926,911	526,725
Time spent watching television	ukb-b-5192	2018	European	9,851,867	437,887
Time spent using computer	ukb-b-4522	2018	European	9,851,867	360,895
Time spent driving	ukb-b-3793	2018	European	9,851,867	310,555

**Table 2:** Basic information about the GWAS database of the outcome.

Outcome	GWAS-ID	Year	Population	Number of SNPs	Sample size
Cardiovascular disease	finn-b-I9_CVD	2021	European	16,380,466	ncase: 111,108 ncontrol: 107,684
Coronary artery disease	ebi-a-GCST005195	2017	European	7,934,254	ncase: 122,733 ncontrol: 424,528
Heart failure	ebi-a-GCST009541	2020	European	7773,021	ncase: 47,309 ncontrol: 930,014
Atrial fibrillation	ebi-a-GCST006414	2018	European	33,519,037	ncase: 60,620 ncontrol: 970,216
Ischemic stroke	ebi-a-GCST006908	2018	European	8,296,492	ncase: 34,217 ncontrol: 406,111

### 2.3. Selection and validation of single nucleotide polymorphisms (SNPs)

We applied 3 criteria to identify independent SNPs related to SB. First, SNPs with a genome-wide significance threshold of *P* < 5 × 10^−8^ were chosen. Second, pairwise linkage disequilibrium was employed to assess the independence of the chosen SNPs. SNPs situated at a distance of 1000 kb apart were chosen, and those in linkage disequilibrium (*r*^2^ ≥ 0.001) were removed.^[18]^ Third, instrument strength was evaluated using the *F*-statistic; SNPs with F > 10 were considered sufficiently strong to minimize weak-instrument bias.^[19]^ If fewer than 10 SNPs were discovered using the criteria, a lower threshold of *P* < 5 × 10^−6^ would be used to satisfy the minimal criteria for MR analysis.^^[20]^^ Furthermore, the PhenoScanner database was used to rule out the effect of possible confounders.^[21]^

### 2.4. Two-sample MR analysis

We employed 4 methods (inverse-variance weighted, MR-Egger, weighted median, weighted mode) in this 2-sample MR study to estimate causal effects between SB and CVD outcomes.^[22]^ Cochran's *Q* test assessed IV heterogeneity, with a *P* < .05 result suggesting heterogeneity. We evaluated horizontal pleiotropy using the MR-Egger intercept test.^[23,24]^ Furthermore, we utilized the leave-one-out sensitivity analysis to determine if a single SNP disrupted the causative impact. TwoSampleMR (0.5.7) packages were used to do all statistical analyses in R software (4.2.3). A value of *P* < .05 was regarded as suggestive significance, and associations with *P* < .0025 (Bonferroni correction *P* = .05/4 × 5) were considered statistically significant.

### 2.5. Power calculation

To ensure adequate statistical power, we estimated the minimum detectable odds ratio (OR) for our MR analyses using the mRnd tool (http://cnsgenomics.com/shiny/mRnd/).^[25]^ Given the sample size and variance explained by the instrumental variables, our study had over 80% power to detect an OR of ≥1.2 for the association between SB and CVD outcomes at an alpha level of 0.05.

## 3. Results

### 3.1. Selection of instrumental variables

Based on the screening criteria, we identified 115 SNPs for leisure screen time, 112 SNPs for time spent watching TV, and 82 SNPs for time spent using computer, all using a threshold of *P* = 5 × 10^‐8^. However, for time spent driving, a more relaxed threshold of *P* = 5 × 10^‐6^ was applied, resulting in the identification of 59 SNPs. After accounting for potential confounders, we excluded 17, 64, 39, and 8 SNPs, respectively. The final set of SNPs included in the analysis was 98 for leisure screen time, 48 for time spent watching TV, 43 for time spent using computer, and 51 for time spent driving. The detailed information on the SNPs included in the MR analysis and those considered as potential confounders can be found in Tables S1–S8, Supplemental Digital Content, https://links.lww.com/MD/P974.

### 3.2. The causal effect of cardiovascular disease

**Figure 1.**
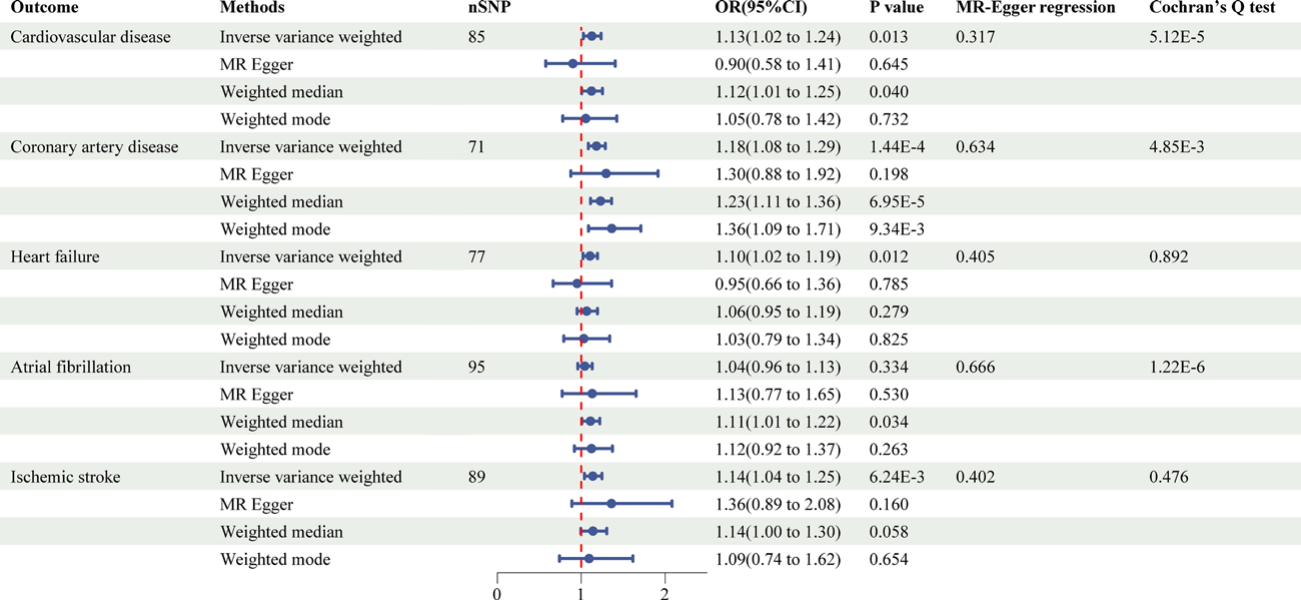
Causal associations between leisure screen time and CVD outcomes. CVD = cardiovascular disease.

**Figure 2.**
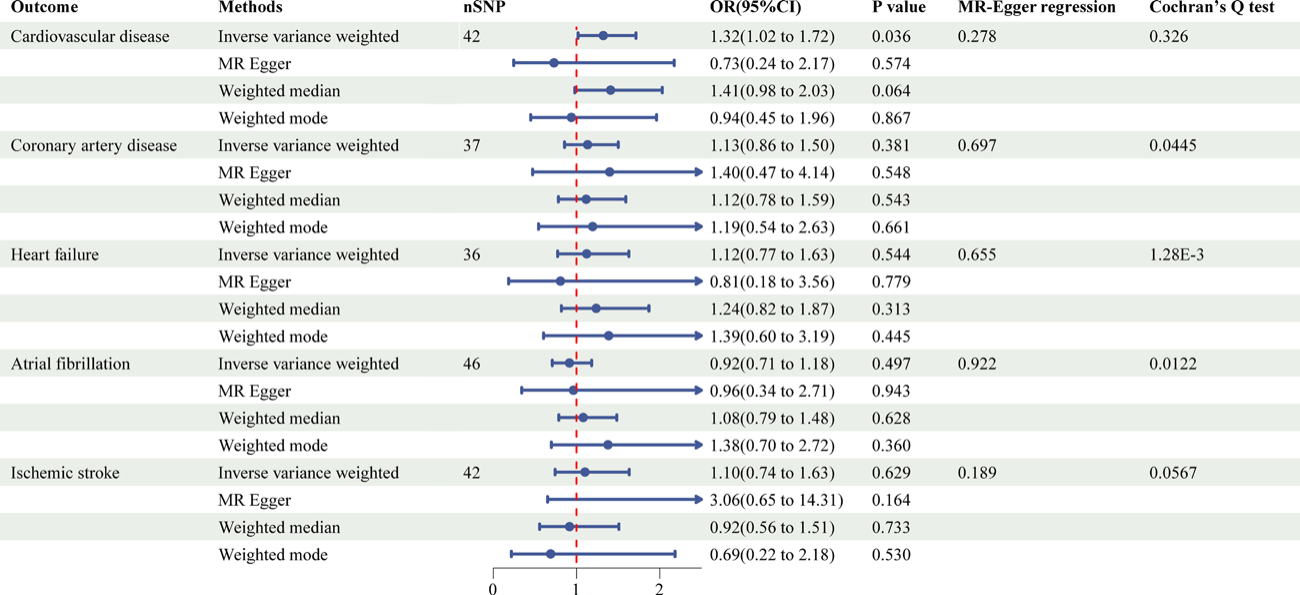
Causal associations between time spent watching TV and CVD outcomes. CVD = cardiovascular disease.

**Figure 3.**
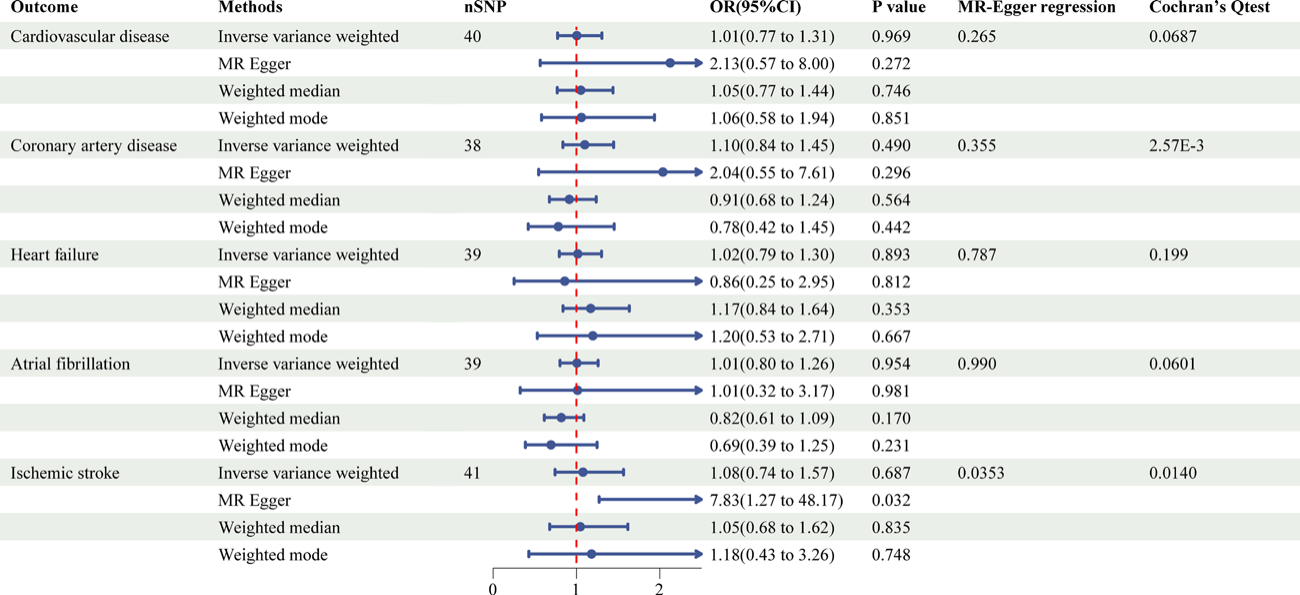
Causal associations between time spent using computer and CVD outcomes. CVD = cardiovascular disease.

**Figure 4.**
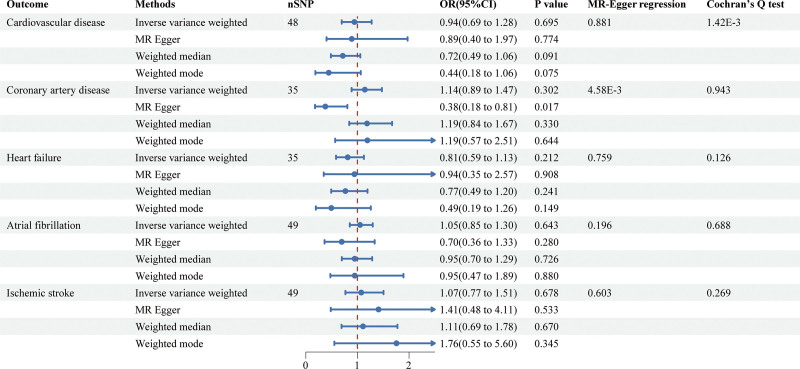
Causal associations between time spent driving and CVD outcomes. CVD = cardiovascular disease.

The 2-sample MR study using 4 methods for the association of LST, time spent watching TV, using computer, and driving with cardiovascular disease was shown in Figures 1–4 and Figure S1, Supplemental Digital Content, https://links.lww.com/MD/P973.

LST showed a significant positive association with CVD (inverse-variance weighted [IVW]: OR = 1.13, 95% confidence interval [CI]: 1.02–1.24, *P* = .013), CAD (IVW: OR = 1.18, 95% CI: 1.08–1.29, *P* = 1.44E^‐4^), HF (IVW: OR = 1.10, 95% CI: 1.02–1.19, *P* = .012), and IS (IVW: OR = 1.14, 95% CI: 1.04–1.25, *P* = 6.24E^‐3^). Notably, the association with CAD remained statistically significant after multiple testing corrections, suggesting a particularly robust relationship. Additionally, time spent watching television was significantly associated with CVD (IVW: OR = 1.32, 95% CI: 1.02–1.72, *P* = .036). In contrast, no significant causal relationships were observed for time spent using computer or driving on any CVD outcomes.

### 3.3. Sensitivity analysis validation

To assess the robustness of our findings, we conducted comprehensive sensitivity analyses using multiple complementary approaches. The primary IVW results were supported by consistent directional effects across MR-Egger, weighted median, and weighted mode methods, although statistical significance was not uniformly achieved. Cochran's *Q* test revealed significant heterogeneity for several associations (*P* < .05), prompting the use of random-effects IVW models to account for this variability.

Evaluation of potential pleiotropic effects through MR-Egger intercept tests showed no evidence of directional pleiotropy, with the exception of the using computer-IS association (*P* = .035) and driving-CAD association (*P* = 4.58E^‐3^). This suggests that our results were generally not biased by horizontal pleiotropy. The stability of our effect estimates was further confirmed by leave-one-out analyses, which demonstrated that no single genetic variant disproportionately influenced the results (Figure S2, Supplemental Digital Content, https://links.lww.com/MD/P973).

Visual inspection of scatter plots (Figure S3, Supplemental Digital Content, https://links.lww.com/MD/P973) provided additional confirmation of the linear relationships between exposures and outcomes. The concordance of effect directions across different analytical methods, combined with these sensitivity analyses, strengthens confidence in the validity of our primary findings regarding the differential cardiovascular effects of specific sedentary behaviors.

### 3.4. Power calculation

We performed statistical power calculations for each MR analysis using an expected OR of 1.20, a significance level of 0.05, and the proportion of variance (*R*²) explained by the genetic instruments for each exposure. The outcome sample sizes ranged from 218,792 (cardiovascular disease) to 1,030,836 (atrial fibrillation). Given the relatively large sample sizes and adequate *R*² values for all 4 sedentary behavior traits (LST, time spent watching television, using computer, and driving), the calculated statistical power for detecting the expected causal effect was 100% across all exposure–outcome pairs. These results indicate sufficient power to detect modest causal associations in our study. Detailed statistical results are provided in Table S9, Supplemental Digital Content, https://links.lww.com/MD/P974.

## 4. Discussion

This MR study systematically evaluated the causal associations between 4 sedentary behaviors and cardiovascular diseases. The results demonstrated that LST was significantly positively associated with multiple cardiovascular outcomes, including CVD, CAD, HF, and IS. Notably, the association with CAD remained statistically significant after multiple testing corrections, suggesting particularly robust evidence for this relationship. Among specific behaviors, only time spent watching television showed a significant causal effect on CVD risk, whereas no significant associations were observed for using computer or driving with any cardiovascular outcomes.

Our findings are broadly consistent with prior research on the relationship between SB and CVD. A large prospective cohort study of 490,966 participants reported that increased television viewing time was associated with higher risks of CVD outcomes, including CVD incidence, CVD mortality, HF, and stroke.^[26]^ Similarly, a 2019 meta-analysis of more than 900,000 individuals found that longer durations of TV watching were significantly associated with to elevated CVD risk.^[27]^ Furthermore, a comprehensive meta-analysis comprising 184 studies concluded that SB increases the risk of cardiovascular disease, while physical activity mitigates this risk and improves lipid profiles.^[28]^

In support of our MR results, a recent MR study using LST as the exposure demonstrated that genetic predisposition to SB was significantly associated with an increased risk of CVD, particularly hypertensive disease, CAD, and cerebrovascular disease.^[29]^ Another MR analysis, which included 3 distinct sedentary behavior exposures, also found that prolonged leisure television viewing constituted a significant risk factor for CAD.^[30]^ However, 2 earlier MR studies reported null associations between SB and CVD outcomes.^[31,32]^ Notably, both studies derived their genetic instruments from the GWAS conducted by Doherty et al in 2018,^[33]^ which did not differentiate between subtypes of sedentary behavior. Moreover, the SNPs identified in that GWAS were entirely distinct from those used as instrumental variables in our study, which may partially explain the discrepancies in results. Unlike previous MR analyses, our study systematically integrates multiple sedentary behavior phenotypes and a broad range of cardiovascular endpoints. This comprehensive approach enhances the robustness and generalizability of our findings and provides a more nuanced understanding of the causal relationship between sedentary behavior and cardiovascular health.

Interestingly, only LST and television viewing—among the four sedentary behaviors evaluated—were associated with specific CVD outcomes, suggesting that context-specific sedentary behaviors may differentially affect health outcomes. Consistent with this, a systematic review reported no association between occupational sitting and adverse health outcomes compared with television viewing. ^[11]^ An Australian study examined associations of sitting time in 4 contexts (occupational, transport, TV-viewing, and leisure-time computer use) with cardiometabolic risk and found that occupational sitting was less harmful and TV-viewing tended to be more detrimental.^[34]^ Similarly, another study discovered that watching TV for more than 4 hours per day increased the risk of CVD events and all-cause mortality, while occupational sitting did not affect these outcomes.^[35]^ Although the underlying mechanisms remain unclear, these findings are consistent with our results. Thus, future research should focus on the context-specific relationships between sedentary behaviors and CVD outcomes.

Previous studies have provided substantial evidence to identify biologically plausible mechanisms for the link between SB and CVD. SB may influence CVD via the following biological pathways. First, SB, particularly excessive television viewing, may raise the likelihood of obesity and diabetes, which are major risk factors for CVD.^[36–38]^ Second, prolonged sitting may lead to endothelial dysfunction via a reduction in shear stress, thereby contributing to atherosclerosis development.^[39,40]^ Third, SB may reduce muscle mass, lower insulin sensitivity, and increase the risks of physical dysfunction.^[41]^ However, the relationship between SB and CVD still needs more in-depth research.

The main strength of this study is its MR design, which eliminated confounding and reverse causation bias. Another advantage is the refinement of SB and the broad spectrum of CVD endpoints evaluated. Nevertheless, it is important to remember that this study has several drawbacks. First, only the relationship between SB and CVD outcomes was investigated. However, the GWAS data only covered the population with SB, and no information on the duration of SB was provided. The dose effect of SB on CVD outcomes was not investigated in the study. Second, the GWAS data from the UK Biobank and FinnGen consortium were all gathered from people of European ancestry, which may limit the applicability of our findings to other racial groups. Third, our analysis was constrained by the specificity of the SB phenotypes available in the GWAS data. The genetic instruments were based on self-reported behaviors, including television viewing, computer use, and driving time, which may not fully capture total sedentary exposure or posture-specific measures. This phenotypic imprecision could lead to exposure misclassification, potentially biasing our effect estimates toward the null. Fourth, partial sample overlap exists between the exposure and outcome GWAS datasets, primarily due to the inclusion of UK Biobank participants in both. This overlap may slightly inflate the causal effect estimates; therefore, the results should be interpreted with appropriate caution. Finally, although there was no indication of pleiotropic effects in our investigation, it is impossible to completely rule out the possibility of directional pleiotropy having an impact. Collectively, this is the first investigation using 2 -sample MR to comprehensively evaluate the relationship between SB and CVD outcomes.

From a clinical and public health perspective, our findings support current guidelines encouraging reduced screen time and increased physical activity as part of cardiovascular disease prevention. While MR estimates are not directly translatable into clinical thresholds, the observed association between genetically predicted LST and television watching and increased CVD risk underscores the potential health benefits of behavioral interventions targeting specific sedentary behaviors.

## 5. Conclusion

This MR study found that longer leisure screen time and time spent watching television were causally associated with higher risks of several cardiovascular diseases, especially coronary artery disease. In contrast, no clear causal links were observed for computer use or driving. These findings suggest that reducing passive screen-based sedentary behaviors may help lower the risk of cardiovascular diseases. From a public health perspective, targeted interventions to limit leisure screen time could contribute to cardiovascular disease prevention at the population level.

## Acknowledgments

We sincerely thank the researchers in the MiBioGen, UK Biobank, and other consortia or studies, and all the researchers who worked on the data collection.

## Author contributions

**Conceptualization:** Qing Hao.

**Data curation:** Ye Cao.

**Formal analysis:** Ye Cao, Qing Hao.

**Investigation:** Ye Cao, Qing Hao.

**Methodology:** Ye Cao, Qing Hao.

**Software:** Nan Xia.

**Supervision:** Nan Xia, Yi Feng.

**Validation:** Nan Xia, Yi Feng.

**Visualization:** Nan Xia.

**Writing – review & editing:** Yi Feng.

**Writing – original draft:** Ye Cao, Qing Hao.

## Supplementary Material


