# Supplementary material for: Exploring the causal relationship between sedentary behavior and cardiovascular disease: A two-sample Mendelian randomization analysis
Source: Medicine (Baltimore). 2025 Sep 12;104(37):e44543. doi: 10.1097/MD.0000000000044543 (PMC12440458; doi:10.1097/MD.0000000000044543)

Figure S1 Forest plots of the association of SBs with CVD outcomes.


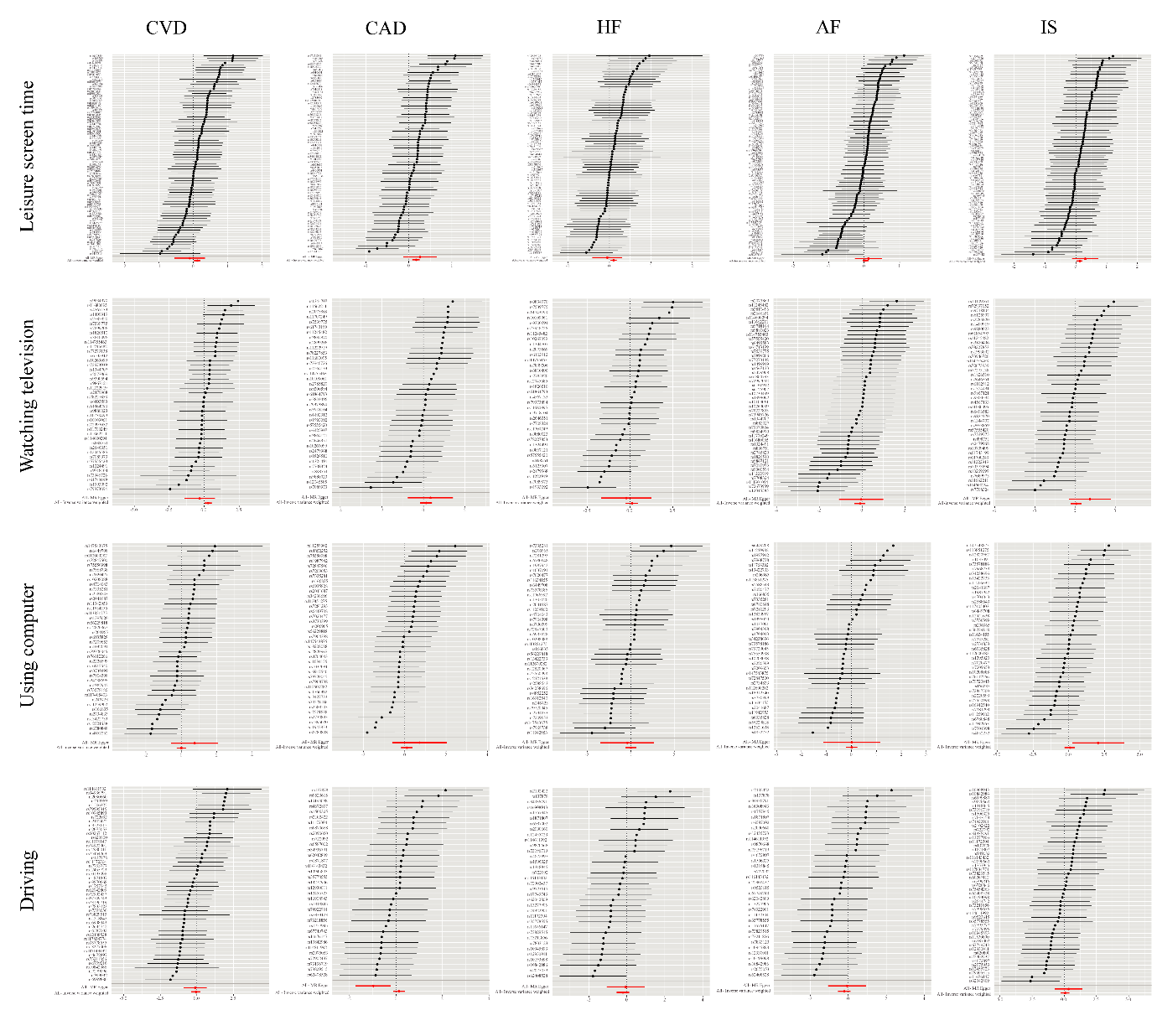


Figure S2 The leave-one-out sensitivity analysis of SBs with CVD outcomes.


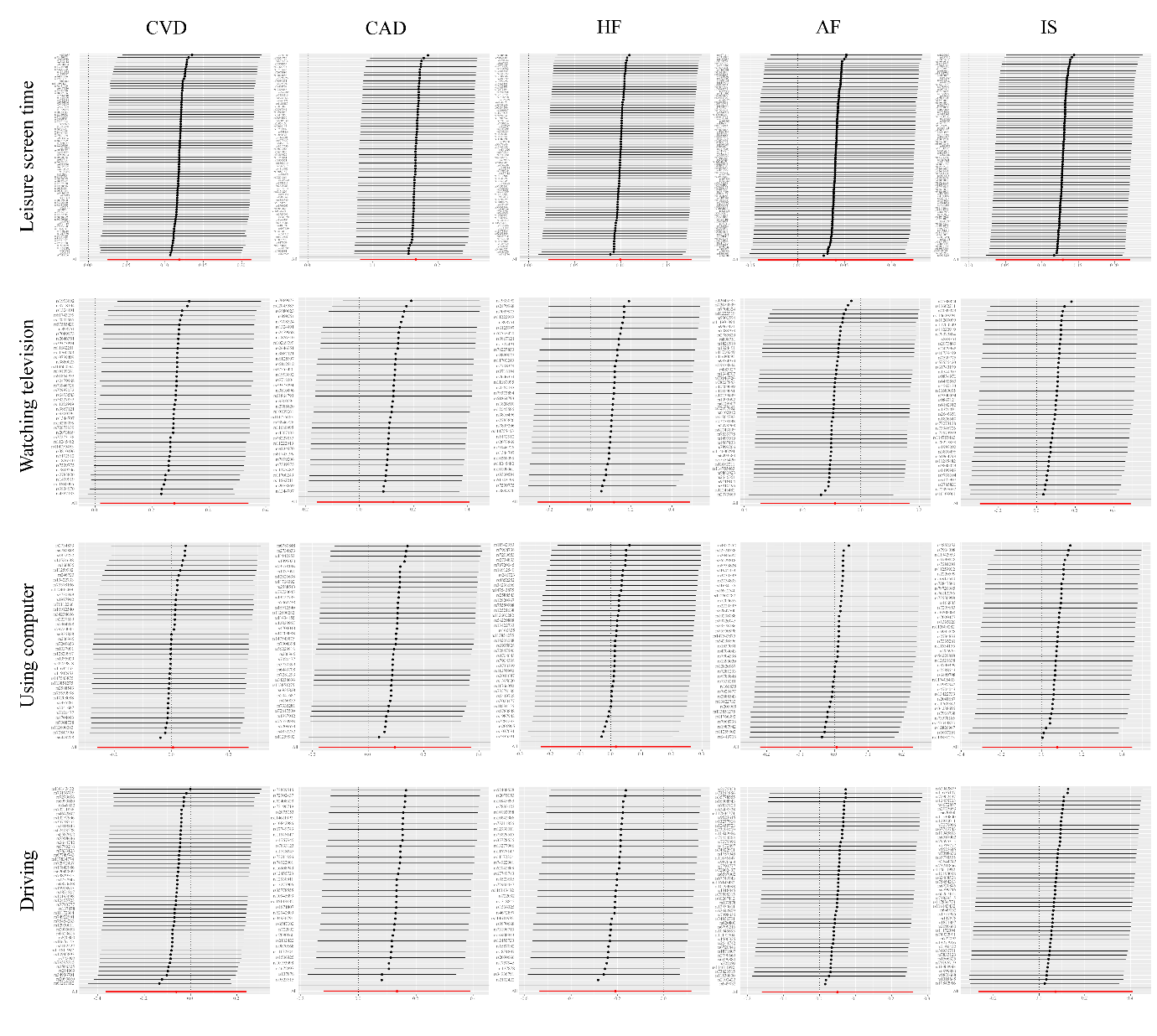


Figure S3 Scatter plots of the association of SBs with CVD outcomes.


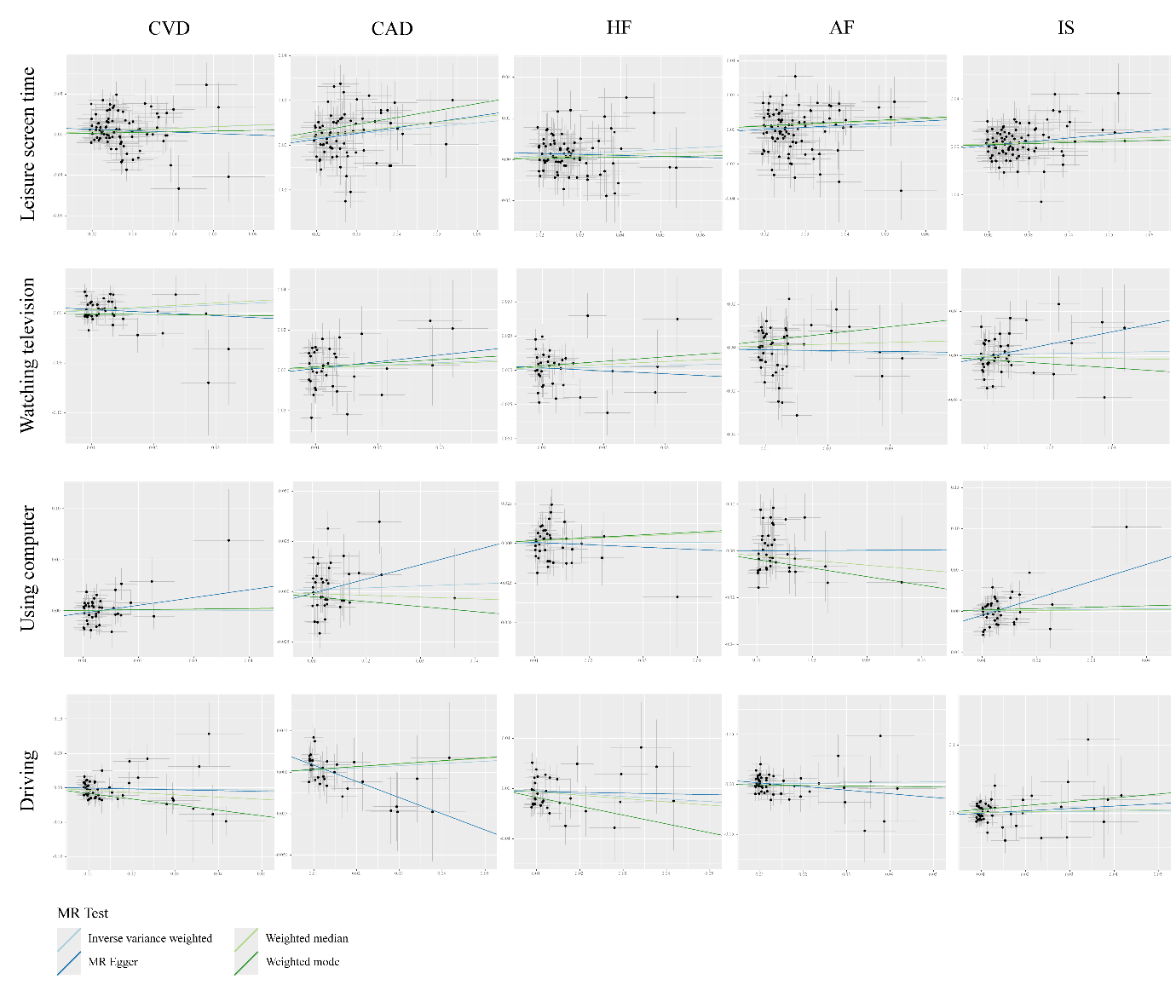

Supplement: Supplementary file 2 [file medi-104-e44543-s002.docx]
